# Sialylation and Muscle Performance: Sialic Acid Is a Marker of Muscle Ageing

**DOI:** 10.1371/journal.pone.0080520

**Published:** 2013-12-11

**Authors:** Frank Hanisch, Wenke Weidemann, Mona Großmann, Pushpa Raj Joshi, Hans-Jürgen Holzhausen, Gisela Stoltenburg, Joachim Weis, Stephan Zierz, Rüdiger Horstkorte

**Affiliations:** 1 Department of Neurology, Martin-Luther-University Halle-Wittenberg, Halle (Saale), Germany; 2 Institute of Physiological Chemistry, Martin-Luther-University Halle-Wittenberg, Halle (Saale), Germany; 3 Institute of Cell and Neurobiology, Charité, Universitätsmedizin Berlin, Berlin, Germany; 4 Institute of Neuropathology, RWTH Aachen University and JARA Brain Translational Medicine, Aachen, Germany; 5 Institute of Pathology, Martin-Luther-University Halle-Wittenberg, Halle (Saale), Germany; University of Leipzig, Germany

## Abstract

Sialic acids (Sia) are widely expressed as terminal monosaccharides on eukaryotic glycoconjugates. They are involved in many cellular functions, such as cell–cell interaction and signal recognition. The key enzyme of sialic acid biosynthesis is the bifunctional UDP-N-acetylglucosamine-2-epimerase/N-acetylmannosamine kinase (GNE), which catalyses the first two steps of Sia biosynthesis in the cytosol. In this study we analysed sialylation of muscles in wild type (C57Bl/6 *GNE* ^+/+^) and heterozygous GNE-deficient (C57Bl/6 *GNE* ^+/−^) mice. We measured a significantly lower performance in the initial weeks of a treadmill exercise in C57Bl/6 *GNE* ^+/−^ mice compared to wild type C57Bl/6 *GNE* ^+/+^animals. Membrane bound Sia of C57Bl/6 *GNE* ^+/−^ mice were reduced by 33–53% at week 24 and by 12–15% at week 80 in comparison to C57Bl/6 *GNE* ^+/+^mice. Interestingly, membrane bound Sia concentration increased with age of the mice by 16–46% in C57Bl/6 *GNE* ^+/+^, but by 87–207% in C57Bl/6 *GNE* ^+/−^. Furthermore we could identify specific morphological changes in aged muscles. Here we propose that increased Sia concentrations in muscles are a characteristic feature of ageing and could be used as a marker for age-related changes in muscle.

## Introduction

Sialic acid (Sia) is a 9 carbon monosaccharide, which is found on the terminal position of most glycans structures of glycoproteins. There it is involved in the stability, turnover und function of glycoproteins. Sia is synthesized in the cytosol in four steps from UDP-GlcNAc. The key enzyme of the Sia biosynthesis is the ubiquitously expressed, bifunctional enzyme UDP-*N*-acetylglucosamine-2-epimerase/*N*-acetylmannosamine kinase (GNE) [1], [2]. GNE catalyses the first two rate limiting steps in the biosynthesis of *N*-acetylneuraminic acid (Neu5Ac), one major member of the Sia family, which consists of more than 50 members (for review see [3] and [4]). Mutations in *GNE* can lead to the rare autosomal-recessive GNE- myopathy (also known as HIBM2; OMIM 600737). It is characterized clinically by adult-onset, slowly progressive, initial distal muscle weakness and atrophy (quadriceps-sparing), and pathologically by the presence of intranuclear tubulo-filamentous inclusions, cytoplasmic rimmed vacuoles, and myopathic changes without evidence of necrosis, inflammation, or oxidative changes [5]–[12].

Under physiological conditions, GNE is expressed in all tissues, including muscle and essential for embryonic development [13], [14]. Inactivation of GNE by homologous recombination (*GNE* ^−/−^, murine C57BL/6 background) results in a drastic reduction of sialylation of embryonic cells and in embryonic lethality at day E8.5 [13]. An overall reduction of about 25% in membrane bound sialic acid was observed in various organs of heterozygous GNE knockout mice (C57BL/6 *GNE^+^* ^/−^) [15]. Interestingly, heart and skeletal muscle were the tissues with the lowest membrane bound sialic acid content in both *GNE^+^* ^/−^ and wild type mice. It is well established that sialylation determine the half-live ( = age) and turnover of glycoproteins. Aged and desialylated glycoproteins in the blood are recognized by the asialoglycoprotein receptor in the liver and removed from the blood stream [16], [17]. Interestingly, there is not only one investigation on sialylation of muscles during ageing. Therefore, the objective of this study was (i) to quantify levels of sialylation of selected muscles during ageing and (ii) to correlate sialylation with morphological and muscle performance analysis in mice. To our surprise we found increased sialylation during ageing and a correlation with low sialylation and muscle performance.

## Results

**Figure 1:**
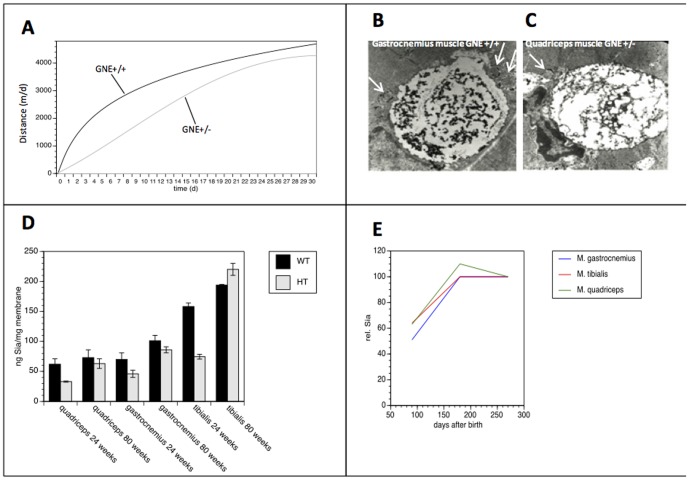
Treadmill exercise in the cohort of the wild type C57Bl/6 *GNE* ^+/+^ (black) and the C57Bl/6 *GNE* ^+/−^ (grey) (**A**). Electron microscopic findings of 80-week old female mice: depicts subsarcolemmal nucleus and perinuclear vacuole containing abundant dense granular material (unlikely to be lipofuscin) in the wild type mouse. Adjacent to the large vacuole are round structures (arrowhead) of sarcoplasmatic tubules, which are confined by double membranes and contain dark granules and in the centre a corpuscule of medium densitiy (gastrocnemius muscle at 16.700×magnification) (**B**). The perinuclear vacuole is surrounded by a single membrane in the heterozygous mouse and displays similar features. Note the two rounded structures in the vicinity containing either membranous structures or dense, granular material (arrowhead) (quadriceps muscle at 16.700×magnification) (**C**). Quantification of sialylation from membrane bound sialic acid in in anterior tibial, gastrocnemic, and quadriceps femoral muscle in wild type C57Bl/6 *GNE* ^+/+^ (black bars) and the C57Bl/6 *GNE* ^+/−^ (gray bars). Values represent means ± 1 SD of three independent experiments (**D**). Relative sialic acid concentrations in different muscles (anterior tibial, gastrocnemic and quadriceps femoral muscle). Sialic acid concentration after 180 days was set to 100% and all other values were expressed in percent of this value. Note that maximal sialic acid concentration was reached after 180 days and no further increase of sialic acid concentration was observed after that time (**E**).

C57Bl/6 *GNE* ^+/−^ mice are vital and we observed no increased mortality of C57Bl/6 *GNE* ^+/−^ mice (or their litter). All mice survived to week 24 or to week 80 dependent on their study group (data not shown). Based on appearance, behaviour, and motor abilities judged under cage conditions there was no obvious difference between C57Bl/6 *GNE* ^+/+^ and C57Bl/6 *GNE* ^+/−^ mice. We then decided to perform treadmill exercise with both C57Bl/6 *GNE* ^+/+^ and C57Bl/6 *GNE* ^+/−^ mice. At age 6 months, C57Bl/6 *GNE* ^+/−^ mice initially showed a dramatically lower performance in treadmill exercise (p<0.05 until day 4) compared to C57Bl/6 *GNE* ^+/+^ (Fig. 1A). The average mean distance per mouse per day (calculated from the sum of all distances of one month) was 3281 m for the C57Bl/6 *GNE* ^+/+^ and 2557 m for the C57Bl/6 *GNE* ^+/−^ (−22% for the C57Bl/6 *GNE* ^+/−^; p<0.05). However, the performance of heterozygous mice improved and between day 21 and day 30 the difference between the two groups diminished to 10–15%. This result possibly indicates delayed adaptive mechanisms in C57Bl/6 *GNE* ^+/−^ mice and prompted us to analyse changes in the skeletal muscle in ageing C57Bl/6 *GNE* ^+/−^ mice.

There was also no difference in the body weight, or skeletal muscle weight of tibial, gastrocnemic, and quadriceps femoral muscles between C57Bl/6 *GNE* ^+/+^ or C57Bl/6 *GNE* ^+/−^ mice at 24 weeks compared to 80 weeks (table 1). However, there was a statistically significant increase in serum creatine kinase levels in mice at 80 weeks compared to mice at 24 weeks in both groups (table 1). We found no pathological changes in muscles of mice at 24 weeks in both C57Bl/6 *GNE* ^+/+^ and C57Bl/6 *GNE* ^+/−^ mice by light and electron microscopy. At week 80, both groups showed a significant increase in central nuclei in all three muscle types investigated and perinuclear acid phosphatase activity, i.e., lipofuscin accumulations, but no other pathological changes in light microscopy (data not shown).

**Table 1:** Comparative data about wild type C57Bl/6 *GNE*+/+ and heterozygous C57Bl/6 *GNE*+/− mice.

	C57Bl/6 *GNE* ^+/+^	C57Bl/6 *GNE* ^+/+^	P-value	C57Bl/6 *GNE* ^+/−^	C57Bl/6 *GNE* ^+/−^	P-value
age (weeks)	24	80		24	80	
number	9	7		10	12	
creatine kinase [µmol/L*s]	191±106 (85–381)	287±156 (93–538)	0.05	231±97 (124–321)	356±97 (103–542)	0.05
total body weight [g ]	25.6±1.4 (23.7–27.8)	24.4	n.s.	28.2±3.5 (23.4–32.6)	24.7	n.s.
anterior tibial muscle weight [mg]	55.5±5.5 (48–62)	45.5±7.1 (37–58)	n.s.	53.1±11.0 (35–66.5)	61.4±8.7 (51–66)	n.s.
gastrocnemius muscle weight [mg]	145.4±16.0 (122–170)	126.6±25.5 (84–157)	n.s.	153.9±21.2 (130–184)	151.9±31.6 (106–194)	n.s.
quadriceps femoris muscle weight [mg]	190.6±21.2 (170–223)	187.9±28.1 (123–217)	n.s.	210.0±41.2 (143–244)	207.3±31.3 (149–253)	n.s.

±SD; range in parentheses; n.s., not significant. Data are mean

*GNE* ^+/+ and^ C57Bl/6 *GNE* ^+/−^ at 24 weeks and at 80 weeks. There was no statistical difference between the parameters in C57Bl/6

Ultrastructural samples analysed at week 80 showed no differences in both groups but age-related changes as tubular aggregates (in male mice only), perinuclear lipofuscin accumulations and vacuoles, and some subsarcolemmal and perinuclear mitochondrial accumulations (in both sexes) (data not shown). In addition, the aged mice occasionally showed peculiar, often perinuclear vacuoles surrounded by membranes containing membranous and dense granular, intensely osmiophilic material (Fig. 1B, C). Sometimes, adjacent to the larger perinuclear vacuoles, round structures of sarcoplasmic tubules were located, which were confined by double membranes and which contained dark granules and in the centre a corpuscule of medium density (Fig. 1B, C).

The quantification of membrane bound Sia , the final biological product of the GNE pathway, revealed highest concentrations in the tibial muscle compared to the quadriceps and gastrocnemic muscles in both C57Bl/6 *GNE* ^+/−^ and C57Bl/6 *GNE* ^+/+^ muscle at 24 and 80 weeks (Fig. 1D). The membrane bound Sia in C57Bl/6 *GNE* ^+/−^ muscles was 33–53% lower at week 24 and 12–15% lower at week 80 compared to C57Bl/6 *GNE* ^+/+^ mice except for the tibial muscle where the Sia concentration was 19% higher in the C57Bl/6 *GNE* ^+/−^ animals. The difference between young C57Bl/6 *GNE* ^+/+^ and C57Bl/6 *GNE* ^+/−^ animals was statistically significant for all three muscles (p<0.05). The membrane bound Sia concentration, however, increased over time by 16–46% in C57Bl/6 *GNE* ^+/+^ and by 87–207% in C57Bl/6 *GNE* ^+/−^. Old animals always showed higher Sia levels than young animals. This applies for both genotypes and all three muscles studied (p<0.05). Furthermore, the increase in Sia concentration was significantly higher in C57Bl/6 *GNE* ^+/−^ than in C57Bl/6 *GNE* ^+/+^ animals (p<0.05). As a result, the differences for Sia levels between old C57Bl/6 *GNE* ^+/−^ and old C57Bl/6 *GNE* ^+/+^ were no longer statistically significant.

To confirm these findings, the Sia concentration was measured in wild type C57Bl/6 *GNE* ^+/+^ mice after 3, 6, and 9 months in the three different skeletal muscles (n = 2 at each time point). Sia concentration after 6 months was set to 100% and all other values were expressed in percent of this value (Fig. 1E). The maximal Sia concentration was reached after 180 days and no further increase of Sia concentration was observed after that time.

## Discussion

We observed a reduced running performance of C57Bl/6 *GNE* ^+/−^ compared to wild type in the treadmill exercise in six months old mice. The further phenotypic examinations of C57Bl/6 *GNE* ^+/−^ mice showed that the Sia concentration in skeletal muscle was decreased up to 53% due to reduced GNE expression. Despite this, no differences regarding long-term mortality, body and muscle weight, and histological features of skeletal muscle compared to age-matched wild type mice were found after 80 weeks. However, the biological consequences *in vivo* are not clear. The mean half life of Sia was shown to be 29 hours and to be shorter than those of the protein portion of the integral plasma membrane glycoproteins [37]. Primary cultured cells isolated from HIBM patients with only 5% GNE activity have a reduction of membrane bound Sia by 70% [28], whereas a GNE−/− knockout clone of BJB-B K20 cells with less than 1% GNE activity had a similar reduction of membrane bound Sia [20].

Furthermore, it is hypothesized that a chronic hyposialylation over a long time leads to the accumulation of morphological defects and, thus, to functional impairment (e.g. HIBM is manifesting in adult age) [21]. However, the Sia concentration in skeletal muscle increased over time in both wild type and heterozygous mice in the present study, even though it was always lower in heterozygous mice compared to controls. However, the difference in muscle Sia content between wild type and heterozygous mice was less pronounced in old compared to young mice reflecting a higher degree of sialylation in old mice and/or a higher need of sialylation in ageing mice. It is noteworthy that the quadriceps showed much lower sialylation levels than the two calf muscles. This is of special interest since HIBM is considered as a quadriceps sparing myopathy. Possibly the low sialylation of the quadriceps is the reason for this phenotype. In addition, there is no increase in sialylation during ageing in the quadriceps. The reason for this distinct feature of the quadriceps is not known. Both Sia content and acidic Sia activity was significantly increased in erythrocytes of patients with diabetes compared to normal individuals [38]. This and our data might suggest either a protective effect in (also prematurely as in diabetes) aged tissue or a decreased turnover.

GNE has been shown to be involved in myogenesis [22] and GNE expression is up-regulated after different types of injury in damaged myofibres as well as in regenerating myofibres with central nuclei [23]. Mild myopathic changes were also a feature of both the aged C57Bl/6 *GNE* ^+/−^ and wild type mice. In addition, the highly sialylated neural cell adhesion molecule (NCAM) was reported to be polysialylated in regenerating myofibres in vivo and in myotubes in vitro suggesting a further role of Sia in muscle regeneration [24], [25]. The loss of Sia and polysialic acid was demonstrated to have effects on the recovery of skeletal voltage-gated sodium channels [26]. Our findings are in line with previous studies that suggest indirectly an increasing sialylation in muscles with age. *In vitro* lectin blot analysis of membrane glycoproteins in the TIG-3 cell line showed that the alpha-2,6-sialylation, but not the alpha-2,3-sialylation, of *N*-glycans decreased in aged cells compared to young cells [27]. Sia levels in smooth muscles of the colon were higher in aged than in young rats whereas the Sia levels in brain and liver decreased over time [28]. Lectin-based proteomic profiling showed drastic increases in Sia and N-glycosylation in the gastrocnemius muscle in 30 month old rats compared to 3 month old rats [29].

It is not clear how changing sialylation contributes to the formation of rimmed vacuoles in HIBM. It was proposed that hyposialylation due to GNE dysfunction in GNE mutations might lead to oxidative stress, protein misfolding, or aggregation [30]. Our findings suggest that moderate hyposialylation is not sufficient to cause specific vacuolar changes in heterozygous mice.

Ultrastructurally we observed typical age-related changes as perinuclear lipofuscin aggregates, tubular aggregates and mitochondrial changes in both aged C57Bl/6 *GNE* ^+/−^ and wild type mice [31]–[36]. In addition we found peculiar perinuclear vacuoles in 18 month old C57Bl/6 mice, which are clearly distinct from classical lipofuscin aggregates but could represent lipofuscin-related structures. Perinuclear nonlipofuscin-like vacuoles in humans are no feature of physiological ageing and are considered pathological. The phenotype of the present C57Bl/6 *GNE* ^+/−^ mouse model emphasizes the difficulties in the application of mouse models to human conditions.

## Materials and Methods

### Animals

All procedures described were approved, and carried out in accordance with the regulation of the Ethics Committee on the Care and Use of Animals of Martin-Luther University Halle-Wittenberg (Germany). C57BL/6 *GNE* ^+/−^ mice and C57BL/6 *GNE* ^+/+^ mice were used (Harlan Laboratories, Germany). The animals were allowed 14 days to acclimatize to the animal care facility, kept at 68–69°F with a relative humidity of 45–60%, a 12 h light: 12 h dark cycle and 10–15 room air changes per hour. Water and food were available without restriction (maintenance diet for mice, Atromin, Germany). The animal model of C57BL/6 *GNE* ^+/−^ mice has been described previously [13]. DNA of mice was collected for genotyping as described previously [13].

### Treadmill exercise

Six months old mice were analysed using cages with integrated running wheels for 28 days. The rotation of 32 running wheels was recorded continuously through an assembly of magnets on the running wheels that triggered reed contacts of the recording circuits. Rotational pulses from all cages were acquired and a data logger cyclically read and reset the 32 counters and saved the raw data including time stamp in a daily sequence of text files. Data acquisition (distance, velocity, and breaks), processing, and presentation were done using LabVIEW (ZMG, Halle, Germany).

### Pathological and morphological analysis

Mice were sacrificed at the ages of 24 weeks ± 2 and 80 weeks ± 2, respectively. Total body weight was measured and blood samples were taken immediately for serum creatine kinase analysis. Tibial, gastrocnemic, and quadriceps femoral muscles were removed, weighed, and snap frozen for light microscopy and membrane preparation, and fixed in 6% buffered glutaraldehyde for electron microscopy. 6 or 10-µm serial cryosections were stained with hematoxylin and eosin (H&E), modified Gomori trichrome, acid phosphatase, cytochrome oxidase (COX) enzyme histochemistry, cavelin 3 antibody (610421, BD Bioscience), and TDP43 antibody (LS C30991, Biozol). Stained sections were visualized using an Axioplan I microscope (Zeiss, Germany) and digitized images were acquired for analysis (Axiovision Rel. 4.8.2, Zeiss, Germany). For electron microscopy, glutaraldehyde-fixed tissue specimens were post-fixed with 1% OsO_4_ in 0.1 M cacodylate buffer containing 50 mM K_3_Fe(CN)_6_ and embedded in epoxy resin and examined with a Philips EM 400 T electron microscope (Aachen) [18] and with a Zeiss TEM 960 electron microscope (Berlin) [19].

### Membrane preparation and sialic acid quantification

Membranes from homogenized mouse tissue were isolated and freeze-dried prior to further analysis as previously described. [15]. Membrane-bound sialic acid was quantified by the periodate/resorcinol method as previously described [15].

### Statistical analysis

All data are presented as means ± SD. All statistical tests were considered significant if P<0.05. Based on sample characteristics, nonparametric statistical analysis was employed (Sigma Stat 3.0, Switzerland).

## Conclusions

C57Bl/6 *GNE* ^+/−^ mice did not develop any specific histological phenotype but showed functional muscular impairment early in life, which appeared to be progressively compensated later in life. The reason for the increased Sia concentration in ageing muscle is not clear but the quantification of cell membrane bound Sia during young adulthood might have prognostic potential and should be studied in more detail. The concentration of Sia in skeletal muscle or possibly in serum or other tissues might serve as a surrogate marker of ageing. Since Sia could have a protective effect the nutritional supplementation should be considered and its effects should be analysed in future studies.
